# *Drosophila* and genome-wide association studies: a review and resource for the functional dissection of human complex traits

**DOI:** 10.1242/dmm.027680

**Published:** 2017-02-01

**Authors:** Michael F. Wangler, Yanhui Hu, Joshua M. Shulman

**Affiliations:** 1 Department of Molecular and Human Genetics, Baylor College of Medicine, Houston, TX 77030, USA; 2 Jan and Dan Duncan Neurological Research Institute, Texas Children's Hospital, Houston, TX 77030, USA; 3 Program in Developmental Biology, Baylor College of Medicine, Houston, TX 77030, USA; 4 Department of Genetics, Harvard Medical School, Boston, MA 02115, USA; 5 Department of Neurology, Baylor College of Medicine, Houston, TX 77030, USA; 6 Department of Neuroscience, Baylor College of Medicine, Houston, TX 77030, USA

**Keywords:** GWAS, *Drosophila*, Functional genomics

## Abstract

Human genome-wide association studies (GWAS) have successfully identified thousands of susceptibility loci for common diseases with complex genetic etiologies. Although the susceptibility variants identified by GWAS usually have only modest effects on individual disease risk, they contribute to a substantial burden of trait variation in the overall population. GWAS also offer valuable clues to disease mechanisms that have long proven to be elusive. These insights could lead the way to breakthrough treatments; however, several challenges hinder progress, making innovative approaches to accelerate the follow-up of results from GWAS an urgent priority. Here, we discuss the largely untapped potential of the fruit fly, *Drosophila melanogaster*, for functional investigation of findings from human GWAS. We highlight selected examples where strong genomic conservation with humans along with the rapid and powerful genetic tools available for flies have already facilitated fine mapping of association signals, elucidated gene mechanisms, and revealed novel disease-relevant biology. We emphasize current research opportunities in this rapidly advancing field, and present bioinformatic analyses that systematically explore the applicability of *Drosophila* for interrogation of susceptibility signals implicated in more than 1000 human traits, based on all GWAS completed to date. Thus, our discussion is targeted at both human geneticists seeking innovative strategies for experimental validation of findings from GWAS, as well as the *Drosophila* research community, by whom ongoing investigations of the implicated genes will powerfully inform our understanding of human disease.

## Abstract (precis)

**Summary:** The use of *Drosophila* for functional investigation of findings from human genome-wide association studies (GWAS) has potential for follow-up of susceptibility signals in more than 1000 human traits.

Over the last two decades, human genome-wide association studies (GWAS) have begun to reveal the genetic risk factors for countless common disorders with complex genetic etiologies (Hardy and Singleton, 2009), including most of the major causes of morbidity and mortality in the developed world. Despite the impressive success rate for discovering disease susceptibility loci, few, if any, results from GWAS have yet to be successfully translated for delivery of new therapies. This is partly explained by challenges implicit in the experimental design; GWAS provide a list of implicated genomic loci from which causal genes must first be identified in order to confidently draw conclusions about biological mechanisms. Another important barrier stems from insufficient communication between the human geneticists who lead GWAS discovery efforts and the researchers best equipped with the tools that are pivotal for further investigations, including simple animal models. Here, we focus on the outstanding and largely untapped potential of the fruit fly, *Drosophila melanogaster*, for functional follow-up of human GWAS. We first provide a basic overview of the methodology behind GWAS, including addressing common misconceptions and highlighting challenges for identifying causal genes. Second, we introduce the *Drosophila* experimental model, covering some key contributions to biomedical science and the powerful genetic tools available for follow-up of GWAS. Third, we present results of cross-species bioinformatic analyses intended as a resource for both human and fly geneticists who are interested in working together to elucidate the genetic mechanisms that underlie complex human traits. Thus, we hope to promote more widespread experimental follow-up of human GWAS in fly models, and thereby accelerate insights leading to novel and much needed therapies.

## GWAS: promise and challenges

The National Human Genome Research Institute Catalog of Published GWAS (Welter et al., 2014; http://www.ebi.ac.uk/gwas/) currently reports 33,004 variant associations with more than 1601 distinct human traits, based on aggregated results from 2224 published GWAS. Each of these human genetic findings is supported by robust statistical evidence. Compared with Mendelian disorders, which are caused by highly penetrant, single-gene mutations, complex genetic diseases are characterized by (1) common polymorphisms with more modest effect sizes; (2) a greater role for polygenicity (additive effects resulting from multiple risk alleles); (3) genetic heterogeneity, in which disease risk is influenced by at least partially non-overlapping loci; and (4) a more prominent contribution of non-genetic factors, including age and environmental exposures. For decades, such disorders evaded genetic dissection until the implementation of GWAS, which have now successfully revealed the presence of risk alleles for innumerable human traits, including coronary artery disease (Lu et al., 2012; Schunkert et al., 2011), multiple sclerosis (De Jager et al., 2009; Sawcer et al., 2011), Alzheimer's disease (Lambert et al., 2013; Naj et al., 2011), and schizophrenia (Ripke et al., 2013; Shi et al., 2011). In fact, as our understanding of human genetics has advanced, many of the distinctions between simple and complex genetic disorders have become less clear-cut. For example, GWAS have now identified common variant modifiers for rare Mendelian disorders (Lee et al., 2015), and reciprocally, next-generation sequencing approaches are beginning to reveal important contributions of rare risk alleles for common diseases (Goldstein et al., 2013). Additional examples have highlighted convergence between studies of rare and common alleles of the same gene, including *SNCA* in Parkinson's disease (Xu et al., 2015), *PCSK9* in dyslipidemia (Teslovich et al., 2010), and *TBX6* in congenital scoliosis (Wu et al., 2015).

**Fig. 1.**
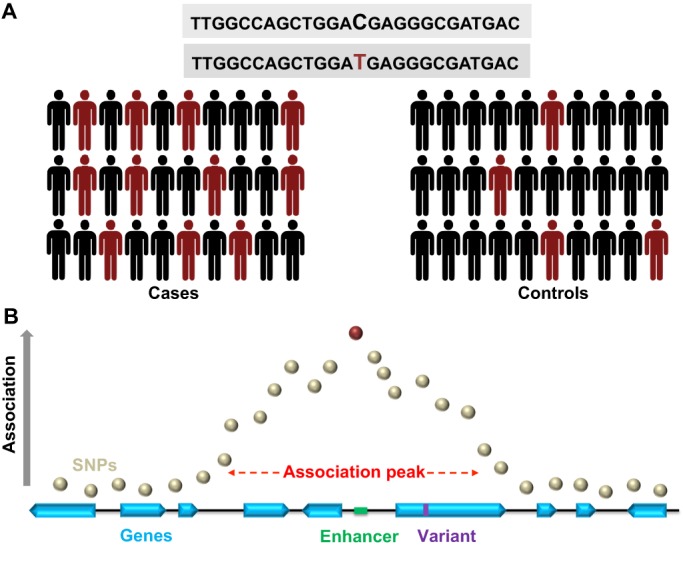
**GWAS experimental design and interpretation.** (A) In human GWAS, subjects with a disease or other trait of interest (Cases) are compared with a cohort of unaffected individuals (Controls). Genome-wide genetic variation is profiled using genotyping arrays, and the resulting frequencies of single nucleotide polymorphisms (SNPs) are evaluated between the groups. In the schematic, a C/T SNP (red) shows a deviation in frequency, with the minor allele, T, being enriched in Cases. Current GWAS examine millions of discrete variants in this manner, and are powered to detect modest frequency differences among cohorts including thousands or tens of thousands of subjects. (B) A schematic ‘association plot’. Such plots are generated based on the results of statistical tests for differences in SNP frequencies versus genomic position, superimposed on the annotated human genome reference. An association signal typically results in a ‘peak’, resulting from regional correlation in genetic variation arising from linkage disequilibrium among SNPs. The SNP showing the strongest association (red) falls within an intergenic region (sequence between genes). In this schematic, a number of candidate causal genes (blue) are implicated, based on several competing considerations. Three genes fall directly under the association peak; however, the top SNP is within an annotated enhancer element (green), raising the possibility of longer-range impact on gene expression outside the immediate region. It is also possible that the causal variant responsible for the regional association has not been directly assayed; for example, a candidate, rare exonic variant (purple) is shown.

In statistical genetics, the association study design simply compares the frequency of an allelic variant between a case and control sample (Balding, 2006) (Fig. 1A). Having controlled for potential confounders between the samples (e.g. ethnic composition, relatedness, or similar factors that might influence genetic makeup), a significant difference in variant frequencies between cases and controls signals the presence of a potential risk or protective allele. An improved understanding of human genomic variation coupled with advances in genotyping technology and statistical methods have enabled association testing on an unbiased, genome-wide scale. In the resulting GWAS, association tests are conducted iteratively for variants sampled across the entire genome. Owing to the block-like structure of correlated genetic variation within genomes (Frazer et al., 2007), current imputation methods allow estimation of millions of genotypes from a much smaller number of directly typed variants. Importantly, given the limitations imposed by commonly employed genotyping arrays and sample sizes, most GWAS conducted to date have been powered for detection of relatively common genomic variants (>1% minor allele frequency). More recently, however, GWAS are also being deployed successfully for analysis of exome-wide genotyping or next-generation sequencing data, highlighting less common or rare variant alleles in population-based case and control cohorts (Francioli et al., 2014; Gudbjartsson et al., 2015; Walter et al., 2015). Besides their application for human genetic investigation, GWAS have further proven to be a powerful method for discovery of genomic variants responsible for complex traits in other species, including *Drosophila*. Indeed, GWAS in flies have successfully identified susceptibility loci for sleep (Harbison et al., 2013), aggression (Shorter et al., 2015), brain size (Zwarts et al., 2015), courtship patterns (Gaertner et al., 2015), sexual characteristics (Takahara and Takahashi, 2015), longevity (Ivanov et al., 2015) and pigmentation (Dembeck et al., 2015), among other phenotypes.

As noted earlier, thousands of susceptibility loci have now been reported for a large diversity of human traits, including many diseases without effective treatments. A central justification for the substantial investment in GWAS has been the potential for advancement of our understanding of disease mechanisms, including insights that could promote development of new therapies. However, the successful discovery of a robust susceptibility signal via GWAS rarely amounts to definitive identification of the responsible gene(s), which is essential for moving from a genetic finding to therapeutic development*.* Instead, GWAS typically identify a marker single nucleotide polymorphism (SNP) that is correlated with an unknown causal genetic variant (Fig. 1B) (Cantor et al., 2010). Therefore, associated SNPs usually identify ‘haplotype blocks’ encompassing up to several hundred kilobases (Wall and Pritchard, 2003) and further fine mapping of such signals to define the responsible genes and variants has proven challenging in most cases (Edwards et al., 2013; Ioannidis et al., 2009). Thus, association peaks from GWAS typically identify a handful of gene candidates, and additional studies are required to determine whether one or more of these are likely to be causal.

Further complicating the picture, it now seems that most GWAS signals are probably the result of regulatory variation – alleles that fall within enhancer elements and therefore impact gene expression – rather than amino acid changes (GTExConsortium, 2015; Nicolae et al., 2010). Because enhancers often act over very long genomic distances, a large number of candidate genes might need to be considered for each associated polymorphism. Nevertheless, ongoing large consortium projects, such as ENCODE (EncodeProjectConsortium, 2004) and GTEx (GTExConsortium, 2015), are enhancing our understanding of non-coding genomic regulatory sequences, allowing improved estimation of the local and more distant changes in gene expression triggered by common variants. Other bioinformatic approaches have been developed to integrate knowledge of genetic pathways with GWAS to test the association between a pathway and a disorder (Cantor et al., 2010; Hindorff et al., 2009). Such efforts promise to simplify the prioritization of candidate genes that could be responsible for associated variants. However, as discussed further below, even once a convincing candidate gene is identified, substantial experimental work is needed to confirm its link to disease susceptibility, including elucidation of the underlying molecular mechanisms.

A frequent criticism of GWAS is that the effect sizes identified for most variants are quite modest. For example, among the ∼22 susceptibility loci identified by the largest Alzheimer's disease GWAS (Lambert et al., 2013), odds ratio estimates for the implicated risk alleles range from 1.1-1.4, whereas the epsilon 4 allele of the apolipoprotein E gene – discovered by linkage analyses completed before the GWAS era – is associated with a threefold increased risk of disease (Corder et al., 1993; Pericak-Vance et al., 1991). However, there are several caveats to consider for interpretation of effect sizes of GWAS. First, as the identified variants are nearly always proxies, they likely yield underestimates for the effect size of the true, but unknown, causal variant. Second, although susceptibility variant effect sizes from GWAS can be underwhelming, their commonality translates to a large contribution to disease risk on a population level. Additionally, individuals frequently harbor multiple such alleles, and corresponding aggregate genetic risk models reveal much stronger combined effects. Third, findings from GWAS hold enormous promise for novel mechanistic insights and potential breakthroughs in treatment, despite the few meaningful advances in clinical risk prediction to date (Manolio, 2013). Notably, evolutionary selective pressures might constrain the frequency of variants with strongly damaging or other functional consequences in human populations, thereby limiting the observed effect sizes of common genomic variants on human traits. By contrast, pharmacological manipulation is not subject to this potential ceiling effect, allowing for more potent therapeutic outcomes. For example, in GWAS of dyslipidemia, common polymorphisms in the HMG-CoA reductase gene have quite modest effects on low-density lipoprotein (LDL) cholesterol levels (Teslovich et al., 2010), whereas treatment with statins, designed to inhibit the encoded enzyme, are powerful LDL-lowering agents taken by millions worldwide for heart disease prevention.

In sum, GWAS have successfully identified thousands of common genomic variants responsible for countless human disease traits. Although such results are supported by robust statistical evidence and represent an enormous opportunity for novel biological insights relevant to disease pathophysiology, the majority of susceptibility loci await functional follow-up, and such work will be essential to leverage GWAS findings for therapeutic advances. Having introduced GWAS, we turn next to exploring the potential for *Drosophila* to accelerate the urgently needed follow-up studies.

## Utilizing *Drosophila* to understand human GWAS signals

**Fig. 2.**
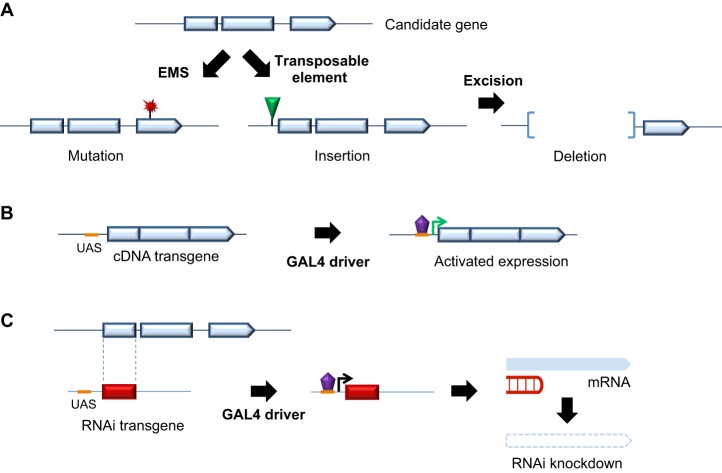
**Strategies for genetic manipulation in *Drosophila*.** (A) Loss-of-function alleles. Large, public *Drosophila* stock collections and laboratories maintain many useful strains for evaluating the consequences of gene loss-of-function. Chemical mutagenesis, such as with ethyl methansesulfonate (EMS), frequently leads to alleles with point mutations (red ‘flash’). Transposable element insertions (green triangle) can also disrupt genes, and further facilitate generation of deletion alleles via imprecise excision. Comprehensive collections of larger chromosomal deletions (deficiencies) covering the entire *Drosophila* genome are also available (not shown). (B) Binary systems for gain-of-function studies. The GAL4-UAS system permits targeted gene activation with precise spatial and temporal control. Transgenic flies harboring a complementary DNA (cDNA) for the gene of interest, under the control of the yeast upstream activating sequence (UAS), can be readily generated or existing lines obtained. Thousands of ‘driver lines’ are also available, which express the yeast GAL4 transcriptional activator (purple) in different tissues and at varying developmental stages. A simple genetic cross brings the two elements together, activating expression of the target gene when the GAL4 transcription factor binds to the UAS sequence. (C) Gene knockdown via RNA-interference (RNAi). Transgenic RNAi strains targeting nearly all *Drosophila* genes are immediately available. When combined with GAL4 driver lines, expression of the short hairpin (under the control of UAS) is activated, leading to degradation of the transcript in a tissue-specific manner.

The vast array of resources and tools that recommend *Drosophila* for functional genomic investigations, including follow-up of human GWAS, has been extensively reviewed (Matthews et al., 2005; Mohr et al., 2014; Ugur et al., 2016; Venken et al., 2011). One distinct advantage is the immediate availability of several large collections of reagents for gene manipulation (Fig. 2). In addition to alleles generated by chemical mutagenesis, transposable element insertions are available for the majority of fly genes (Fig. 2A), including homologs of candidate susceptibility genes from GWAS. Specifically, the *Drosophila* Gene Disruption Project has generated transposon insertion alleles for over two thirds of the organism's protein-coding genes (Bellen et al., 2011). These strains facilitate further genomic manipulations, including the generation of deletion alleles via imprecise excision of transposable elements (Fig. 2A). Collections of deficiency strains tiling nearly the entire *Drosophila* genome provide another valuable resource for reverse genetic studies (Cook et al., 2012; Ryder et al., 2007). Binary expression systems (Fig. 2B) in which a transcriptional activator binds to specific cis-enhancer elements, leading to activation of the adjacent gene, are enormously flexible, popular and powerful research tools. GAL4/UAS, which was co-opted from yeast, is the most widely used binary expression system, and thousands of GAL4 driver lines available from individual labs and public stock collections allow expression of desired target genes, typically cDNA transgenes under control of upstream activating sequence (UAS) sites, in precise spatial and temporal patterns (Brand and Perrimon, 1993). Extensive collections of transgenic RNA-interference (RNAi) lines are also available (Dietzl et al., 2007; Perkins et al., 2015). Under control of the GAL4-UAS system, these RNAi strains permit tissue-specific and/or conditional knockdown of up to 88% of all protein-coding genes (Fig. 2C), facilitating efficient analysis of loss-of-function phenotypes. Information on these and many other useful genetic reagents is available through FlyBase, a central, online annotated resource for *Drosophila* genetics (http://flybase.org; dos Santos et al., 2015; Millburn et al., 2016).

Leveraging substantial genomic conservation and powerful genetic tools outlined above, studies in *Drosophila* have touched nearly all branches of human disease biology (Wangler et al., 2015). This includes insights into mechanisms of congenital renal disease (Weavers et al., 2009), cardiotoxicity from a high-fat diet (Diop et al., 2015), sterol absorption in the gut (Voght et al., 2007) and neuromuscular dysfunction in mucolipidosis (Venkatachalam et al., 2008). *Drosophila* models have enabled powerful mechanistic insights into numerous neurodegenerative disorders (Jaiswal et al., 2012; Lessing and Bonini, 2009; Shulman et al., 2003) including Alzheimer's disease, Parkinson's disease, amyotrophic lateral sclerosis, Huntington's disease and spinocerebellar ataxias (Lessing and Bonini, 2009; Romero et al., 2008; Tsuda et al., 2008, 2005; Vos et al., 2012). Unbiased genetic screens leveraging RNAi reagents in the fly have been deployed to identify conserved regulators of cardiac function (Neely et al., 2010b), pain perception (Neely et al., 2010a) and adipocyte differentiation (Pospisilik et al., 2010). Other screens have highlighted Mendelian disease genes (Yamamoto et al., 2014). For such disorders, *Drosophila* readily facilitates confirmation of variant pathogenicity and elucidation of disease mechanisms (Bellen and Yamamoto, 2015; Shulman, 2015). In the broad scope of investigations using fly models for functional genomics, follow-up of results from human GWAS is still comparatively new. Nevertheless, there is enormous potential for such work, and we highlight below several noteworthy and pioneering examples.

**Fig. 3.**
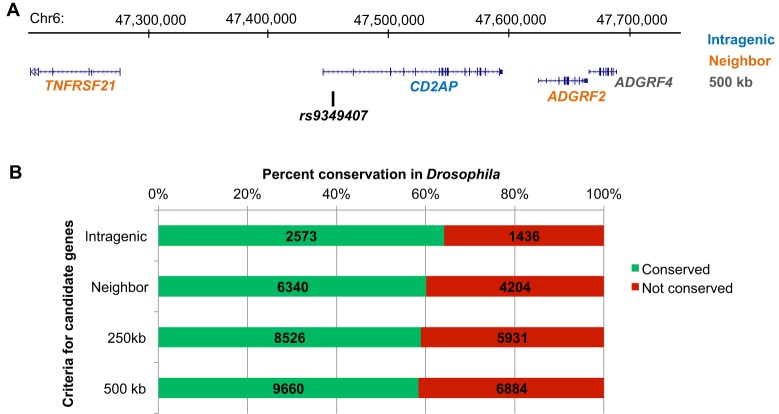
**Identification of GWAS candidate genes and conservation in *Drosophila*.** (A) The Alzheimer's disease susceptibility locus on chromosome 6 identified by the index SNP, *rs9349407* (Naj et al., 2011), is provided as an example to highlight candidate gene selection strategies. For the analyses of gene conservation, we defined four nested criteria for candidate genes. ‘Intragenic’ genes are denoted by an exonic or intronic SNP (blue: *CD2AP*). ‘Nearest neighbors’ additionally include genes immediately proximal and distal to the SNP (orange: *TNFRSF21*, *ADGRF2*). Lastly, a genomic window is defined around the index SNP, extending 125 kb or 250 kb both proximal and distal to define all gene candidates ‘within 250 kb’ or ‘within 500 kb’, respectively (grey: *ADGRF4*). These criteria were applied systematically to all 15,825 SNPs within the NHGRI-EBI GWAS catalog (September 2015 data freeze) to define candidate genes; the full results are detailed in Table S2. (B) Conservation of human susceptibility gene candidates in *Drosophila*, based on the *Drosophila* Integrative Ortholog Prediction Tool (DIOPT, Score ≥2) (Hu et al., 2011). A modest enrichment for conservation was observed for candidate genes defined from intragenic SNPs compared with the other criteria.

Regardless of the experimental model selected, GWAS present unique challenges for functional follow-up investigations, and one immediate obstacle is how to prioritize specific candidate genes for further study based on associated variants. Because most SNPs detected by GWAS are not likely causal variants for disease risk but rather informative markers, it is often not productive to study their direct functional consequences. Furthermore, the non-coding sequences that usually harbor such changes show less conservation than exonic regions, especially in evolutionarily distant species. Instead, implicated SNPs must be mapped to the most promising gene candidates and usually, there are multiple prospects (Fig. 1B, Fig. 3A). One strategy is to leverage medium- to high-throughput screening assays in flies to help refine and prioritize from among several, equally good, candidate genes in order to identify one (or more) worthy of more detailed study. For example, Pendse et al. (2013) studied 38 human genomic regions based on SNPs linked to type 2 diabetes mellitus or related metabolic traits. A 100 kb genomic window centered around each associated SNP identified 130 human gene candidates, of which 71 were sufficiently conserved for follow-up in flies. Orthologous genes were serially targeted using available RNAi transgenic lines and examined for genetic interactions in a fly model relevant to diabetes, based on sucrose-induced toxicity. As illustrated in Fig. 2C, short hairpin RNA sequences homologous to each target gene were activated using a ubiquitously expressed *tubulin*-GAL4 driver line. Using this strategy, 34 human genes were highlighted based on enhancement or suppression of sucrose-induced lethality, following knockdown of their respective gene orthologs. The majority of genomic regions studied had multiple human candidate genes, and in six cases, a single gene was implicated based on the screening assay, allowing refinement of the initial list. Interestingly, in nine other cases, more than one gene in a region showed independent interactions with sucrose toxicity, potentially compatible with the contribution of multiple genes to each human susceptibility signal. Based on more recent GWAS (Bonnefond and Froguel, 2015), more than 90 susceptibility loci have now been implicated in type 2 diabetes, increasing the need for further follow-up studies. Indeed, the vast majority of susceptibility signals identified by human GWAS to date await further fine-mapping efforts to prioritize and confirm the responsible genes.

Where one or more well-conserved gene candidates are strongly implicated, *Drosophila* is an ideal model system for further functional elucidation. The prevailing strategy relies on the key assumption that beyond evolutionary conservation at the sequence level, homologous genes will have conserved functional requirements in humans and in *Drosophila*, leading to similar traits or phenotypes when subject to genetic manipulation*.* In a GWAS for alcohol consumption that included more than 40,000 individuals, the top-associated SNP fell within the intron of the autism-linked *AUTS2* gene, which encodes a neuronal nuclear protein of uncertain function (Schumann et al., 2011). The implicated variant was further related to *AUTS2* expression levels in postmortem human brain samples, consistent with *AUTS2* being a candidate causal gene for the association. In order to complement the largely correlative data with functional evidence, the authors next turned to *Drosophila*, which has a single gene ortholog, *tay*. Ethanol tolerance was enhanced by inactivation of *tay* using transposable element insertional alleles or pan-neuronal knockdown of the gene by RNAi (Schumann et al., 2011). Specifically, *tay* loss-of-function flies were observed to maintain consciousness longer than control animals when exposed to ethanol vapor. In another study focused on identifying risk factors for idiopathic azoospermia in a cohort of more than 9000 men, a similar strategy was employed for follow-up of a strongly suggestive association signal proximal to the gene candidate, *CDC42BPA*. Indeed, knockdown of the fly homolog, *gek*, via RNAi transgene expression in the supporting somatic cells of the fly testis led to impaired sperm maturation and resulting infertility (Hu et al., 2014). Lastly, in a more targeted *Drosophila* follow-up study of a top variant associated with restless legs syndrome (Stefansson et al., 2007), mutant alleles of a conserved homolog of the leading candidate gene, *BTBD9*, were generated via transposon-mediated excision (Fig. 2A), causing motor restlessness and sleep fragmentation that was remarkably reminiscent of the human disorder (Freeman et al., 2012).

Given the success of using *Drosophila* to model neurodegenerative disorders, it is not surprising that such systems have been applied to follow up findings of GWAS in this field. In one early example of this strategy, we used fly transgenic models to test the hypothesis that candidate Alzheimer's disease susceptibility genes modulate neurodegenerative phenotypes induced by expression of the human MAPT protein, responsible for the characteristic neurofibrillary tangle pathology (Shulman et al., 2011). Specifically, MAPT-induced retinal degeneration causes a visible eye phenotype in flies that is modified by knockdown or activation of susceptibility gene homologs. In subsequent work (Chapuis et al., 2013; Moreau et al., 2014; Shulman et al., 2014), we and others discovered that fly homologs of several genes at loci implicated by GWAS, including *BIN1*, *PICALM*, *CD2AP*, *CELF1* and *FERMT2*, can interact with MAPT toxicity *in vivo*, providing mechanistic insight into their potential links with Alzheimer's disease risk in humans. Similarly, GWAS in Parkinson's disease have recently (Nalls et al., 2014) expanded to ∼28 the number of susceptibility loci identified by common genomic variants, and studies in *Drosophila* have shown promise for mechanistic follow-up. Interestingly, results from two recent studies (Ivatt et al., 2014; MacLeod et al., 2013) highlight connections between candidate genes identified by GWAS and causes of Mendelian Parkinson's disease. Consistent with findings in neuronal cell cultures, Macleod et al. found that overexpression of the fly homolog of *RAB7L1* (also known as *RAB29*), a candidate gene in the *PARK16* susceptibility locus, was capable of rescuing dopaminergic neuronal loss and reduced survival induced by LRRK2^G2019S^, which is associated with autosomal dominant familial Parkinson's disease. LRRK2 toxicity was also suppressed by overexpression of the conserved *Drosophila* homolog of *VPS35*, rare variants that also cause dominantly inherited Parkinson's disease. Prior studies in numerous models (including flies) demonstrated that *VPS35* is required for retrograde transport of proteins within the endosomal-lysosomal pathway (Wang et al., 2014). In the second study, Ivatt et al. (2014) performed a genome-wide RNAi screen in *Drosophila* cells to identify genes required for translocation of the Parkinson's disease-associated protein parkin upon mitochondrial damage. Mutation of the parkin (*PARK2*) gene is a common cause of early-onset, autosomal recessive parkinsonism, and studies in *Drosophila* have contributed substantially to our understanding of its putative role in mitochondrial quality control (Haelterman et al., 2014). Interestingly, the cell-based parkin interaction screen identified multiple mediators of lipogenesis, including *SREBF1*, a Parkinson's disease susceptibility candidate gene identified via GWAS. Thus, these studies exemplify how investigation in *Drosophila* can not only link findings from GWAS to informative, disease-relevant biology, but can additionally reveal connections between simple Mendelian and more complex genetic forms of disease.

Despite the opportunities for advancing our mechanistic understanding of many common human diseases with complex genetic etiologies, *Drosophila* certainly has its limitations for follow-up of GWAS. As discussed above, the majority of susceptibility signals identified by GWAS likely point to regulatory variants. However, compared with protein-coding sequences, cross-species evolutionary conservation of genomic regulatory sequence is less well defined, especially between humans and flies. Thus, it is rarely feasible to directly examine the functional consequences of presumed regulatory variants. Instead, *Drosophila* is more appropriate for gene-centric strategies that investigate consequences of directed experimental manipulation of genes on relevant phenotypes. The potential application of this approach depends on gene conservation. Even where conservation of genes is strong, a related question is whether encoded proteins will subserve *conserved functions* in such evolutionary distant species. Thus, as considered systematically below, in order to understand the potential applicability of *Drosophila* for follow-up of human susceptibility loci, we need to understand not only the conservation of candidate susceptibility genes, but whether available evidence supports conserved functional requirements.

## A resource for functional follow-up of GWAS in flies

### Conservation of candidate human susceptibility genes in *Drosophila*

In order to assess the potential of *Drosophila* for follow-up of GWAS – and to directly facilitate future studies – we have undertaken systematic cross-species analyses based on the comprehensive results reported in the National Human Genome Research Institute–European Molecular Biology Laboratory-European Bioinformatics Institute (NHGRI-EBI) GWAS catalog (http://www.ebi.ac.uk/gwas). Selected findings and examples are highlighted below, and the full results are available in supplemental Tables S1-S4. As a starting point, we applied consistent criteria to define candidate human susceptibility genes based on reported SNP associations for each trait. The overall approach is illustrated in Fig. 3A, taking as an example a chromosome 6 SNP, *rs9349407*, discovered in a large GWAS of Alzheimer’s disease risk (Naj et al., 2011). This SNP falls within an intron of the *CD2AP* gene, which encodes an actin-binding and SH3 domain adaptor protein, but additional candidate genes can be defined based on genomic intervals centered around the index variant, the size of which determines the number of implicated genes. In our analyses, we define four nested categories that progressively consider an increasing number of candidate genes at each implicated locus: (1) ‘intragenic’, referring to genes in which the associated SNP falls within an intron or exon (*n*=0-1 genes/SNP); (2) ‘nearest neighbors’, including the genes immediately proximal and distal to the SNP (*n*=2-3 genes/SNP); (3) ‘within 250 kb’, including all genes within a genomic window 125 kb proximal and 125 kb distal to the SNP (*n*≈0-20 genes/SNP); and (4) ‘within 500 kb’, in which the genomic window is extended a further 250 kb proximal and distal to the SNP (*n*≈0-37 genes/SNP). Therefore in our example (Fig. 3A), the *rs9349407* SNP identifies *CD2AP* (intragenic), *TNFRSF21* and *ADGRF2* (nearest neighbors), and lastly *ADGRF4* (within 500 kb). These four levels of criteria were applied to all 15,825 reported SNP associations within the NHGRI-EBI GWAS catalog (September 2015 data freeze), resulting in between 4009 (intragenic) and 16,544 (within 500 kb) total human candidate susceptibility genes, which we consider for the analyses described below. In Table S2, we also make available the comprehensive list of candidates.

Having defined a candidate gene list, we next asked which loci are conserved in *Drosophila*, a key prerequisite for following up a candidate susceptibility gene from GWAS in flies. We took advantage of the published *Drosophila* Integrative Ortholog Prediction Tool (DIOPT) (Hu et al., 2011), which integrates 10 bioinformatic algorithms, to evaluate putative human-fly ortholog pairs. We required that at least two distinct algorithms agree (DIOPT score≥2) for determination of whether a human candidate susceptibility gene is conserved. Although these rather liberal criteria are potentially liable to false-positive calls of homology, they are adequate for our goals to (1) assess potential conservation among large groups of genes and (2) inform selection of genes for follow-up experimental validation, which ultimately is essential to confirm any bioinformatic predictions. Applying these criteria genome-wide, 11,122 out of 20,950 (53%) protein-coding genes in the human genome have a fly ortholog. Based on prior work documenting increased conservation of human genes linked to Mendelian disorders (Fortini et al., 2000; Hu et al., 2011) we hypothesized that on average, susceptibility genes for complex human diseases would also show increased conservation. Indeed, we discovered a modest enrichment (1.2-fold) of cross-species conservation for candidate genes mapped to SNPs discovered in human GWAS. Interestingly, the degree of conservation is strongest (64%, 2573 of 4009 genes) for intragenic genes, whereas enrichment is attenuated somewhat when the criteria are liberalized (58% for genes within 500 kb, 9660 of 16,544 genes) (Fig. 3B). If genes with roles in disease susceptibility (such as those implicated by GWAS) are indeed more likely to be evolutionarily conserved, than this observation might suggest that the genes harboring intragenic associated variants are more likely to be truly causal than candidates mapped at greater distances from SNPs; in other words, cross-species conservation could help to guide fine mapping of causal genes. For all subsequent analyses, we restricted our consideration to candidate susceptibility genes identified by intragenic SNPs.

The human GWAS catalog reports findings from studies of a diverse array of human traits and disease phenotypes, ranging from risk of autism to economic and political preference. We wondered whether all such traits are equally translatable for studies in fruit flies. In order to facilitate comparisons, we categorized each of the 1252 traits based on two separate criteria: (1) target tissue (Table 1) or (2) disease mechanism (Table 2) (full results in Table S1). For target tissue, we considered the cell type and/or organ system that is predominantly impacted by the trait or disease (e.g. heart failure primarily affects the cardiovascular system). However, diseases that affect the same organ systems often have widely divergent genetic mechanisms. Although ischemic stroke, multiple sclerosis, and Parkinson's disease similarly affect the central nervous system, distinct mechanisms are implicated (e.g. vascular, immunological and/or inflammatory, and neurodegenerative etiologies, respectively), implying that the underlying genes – and resulting extent of evolutionary conservation – might differ. The principle of aggregating traits based on common mechanism has been leveraged for the discovery of shared genetic risk factors, formally known as pleiotropy (Solovieff et al., 2013). For example, a GWAS integrating data on more than 30,000 subjects with autism, attention deficit-hyperactivity disorder, bipolar disorder, major depressive disorder and schizophrenia identified 38 candidate genes (Smoller et al., 2013), of which 27 (73.0%) are conserved in the *Drosophila* genome.

**Table 1.**
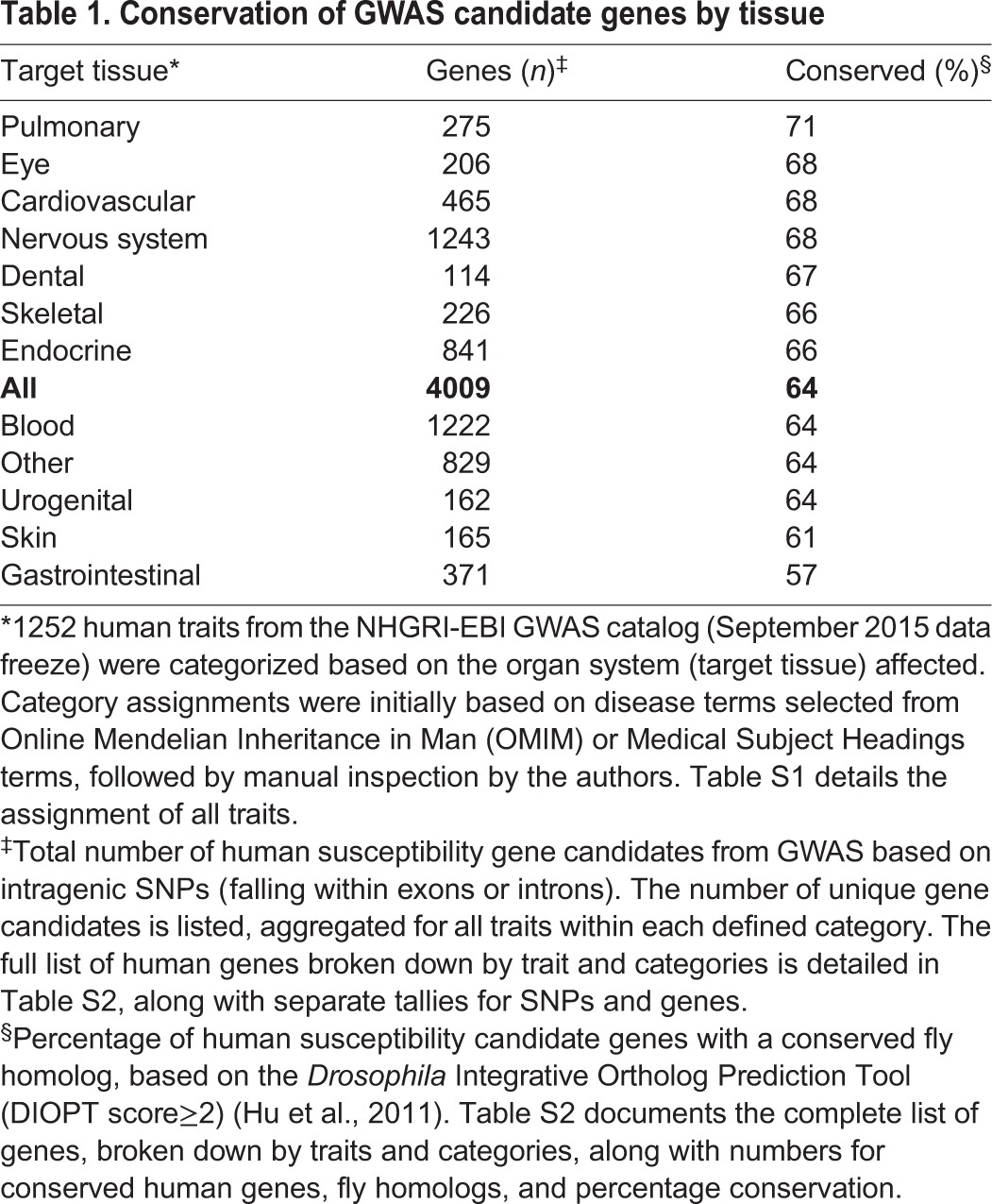
**Conservation of GWAS candidate genes by tissue**

**Table 2.**
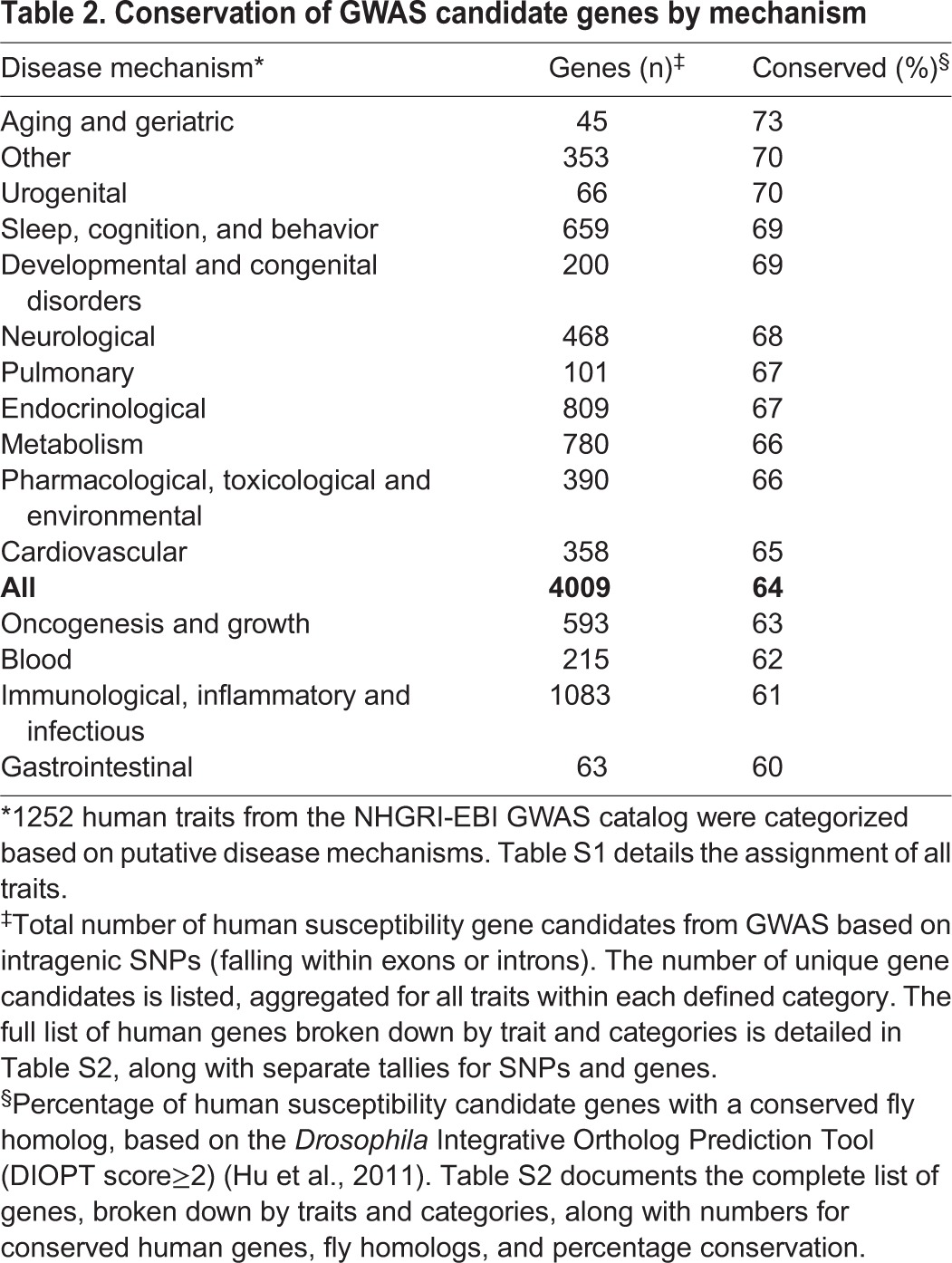
**Conservation of GWAS candidate genes by mechanism**

We therefore examined whether particular human phenotypes or categories are more or less amenable to study in *Drosophila*, again using conservation of implicated genes from GWAS as a benchmark (Tables 1, 2; Table S2). One potential caveat for interpretation of these analyses is that not all human traits (or trait categories) have been interrogated with the same intensity. Variation in the number of studies and sample sizes employed impact the statistical power for discovery of susceptibility loci. Nevertheless, based on either target tissues or mechanisms, all human trait categories showed evidence of increased conservation compared with the genome-wide average (53%, above). However, as shown in Tables 1 and 2, we observed substantial variation (range: 57-73%) in the degree of conservation for implicated genes depending on the specific category. For example, when compared with the composite set of all genes nominated by GWAS, several target tissue categories showed increased conservation (e.g. pulmonary, eye, cardiovascular, nervous system) whereas others showed reduced conservation (e.g. blood, urogenital, skin, gastrointestinal). Interestingly, the alternative classification scheme based on disease mechanisms generally increased conservation within and across categories; gastrointestinal disorders remained the lowest ranked group. As expected, even within categories there can be significant variation in conservation based on specific traits. For example, among all nervous system traits with at least 20 gene candidates identified, Alzheimer's disease cognitive decline was the trait with the greatest conservation (80%, 32 out of 40 gene candidates).

In sum, candidate susceptibility genes nominated by human GWAS generally show increased conservation in *Drosophila* when compared with the average level of conservation observed for all human genes. In addition, the degree of conservation depends on the specific trait, and is influenced by both the target tissue and underlying putative disease mechanisms. Our comprehensive analytic results (Table S2) will allow human and fly geneticists to infer the extent to which *Drosophila* is amenable for follow-up of specific disease traits and/or categories, and provide an accessible catalogue of the fly genes that are homologous to human susceptibility gene candidates identified by published GWAS and thereby represent a high priority for functional studies.

### Expression of homologs of human susceptibility genes

**Fig. 4.**
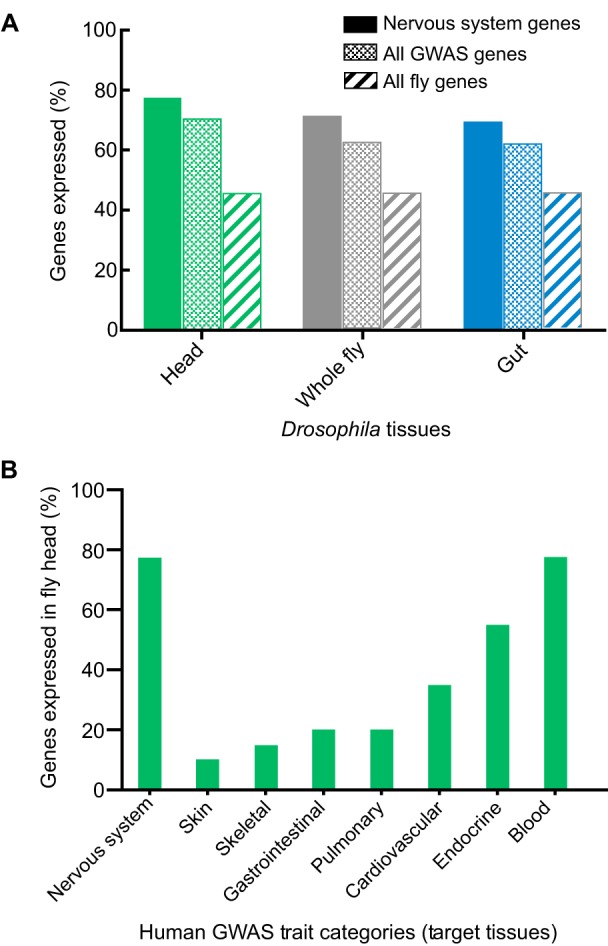
**Expression of GWAS gene homologs in *Drosophila* tissues.** (A) Fly homologs of human susceptibility gene candidates (based on intragenic SNPs) from nervous system disorder GWAS were evaluated for expression in *Drosophila* tissues [reads per kilobase of transcript per million (RPKM) ≥2], including adult head (green), whole fly (gray), gut (blue). Nervous system traits and candidate genes were aggregated based on the ‘target tissue criteria’, as defined in the text and Tables S1 and S2. Tissue-specific RNA-seq data generated by the modEncode consortium was downloaded from FlyBase (ftp://ftp.flybase.net/releases/current/precomputed_files/genes/gene_rpkm_report_fb_2015_03.tsv.gz). 77% of fly homologs of nervous system GWAS candidate genes (solid bars) were expressed in the fly head, a modest increase from those expressed in the whole fly (72%) or gut (69%). For comparison, expression data is also shown for all candidate genes from GWAS (stippled bars) and all fly genes (striped bars). In general, fly homologs of genes identified by GWAS tend to be expressed at higher levels in *Drosophila* tissues. Full results for all target tissue and disease mechanism categories for GWAS across all available *Drosophila* tissues are included in Table S3. (B) The fly homologs of nervous system gene candidates identified by GWAS are more likely to be expressed in the adult *Drosophila* head than homologs for most other target tissue categories.

In addition to gene or protein sequence conservation, the tissue-specificity of expression patterns (or lack thereof) is highly relevant when considering investigation of a candidate human disease gene in an experimental model organism. For example, when confronted with a novel brain-expressed candidate gene for Parkinson's disease, well-conserved in flies, it might be important to ask whether the gene is consistently expressed in the *Drosophila* nervous system. As well as supporting further functional studies in flies, a positive answer could also increase confidence that the selected candidate gene is truly causal. In order to address this systematically, we leveraged publicly available, high-throughput *Drosophila* gene expression data (Brown et al., 2014) to determine the levels and potential tissue-specificity for *Drosophila* homologs of candidate susceptibility genes from human GWAS. Similar analyses can be readily performed using the publicly available *Drosophila* Gene Expression Tool (DGET, http://fgr.hms.harvard.edu/dget). We initially focused on nervous system traits, and found that 77% of fly homologs for human neurological disorder susceptibility genes were expressed in the adult fly head. Furthermore, expression of these genes seemed somewhat more likely to be detected in the fly head than in the fly digestive system (69%), or whole animal (72%) (Fig. 4A), consistent with the hypothesis that homologs of genes associated with human nervous system disorders, show a relative, albeit modest, tissue specificity in flies. In addition, the fly homologs of candidate genes expressed in the human nervous system are somewhat more likely to be expressed in the adult fly head than are homologs of candidate genes nominated by other human target tissue categories (Fig. 4B; full results in Table S3). One potential caveat comes from the finding that the fly homologs of human candidate susceptibility genes from GWAS tend to be expressed at higher levels not only in the fly head, but also across a broad range of other *Drosophila* tissues for which data are available (Fig. 4A; Table S3). Indeed, strongly conserved *Drosophila* genes, including the homologs of most candidate susceptibility genes from GWAS, seem to be expressed more widely and robustly, consistent with conclusions from our recent work (Y.H., unpublished observation). Given that most signals in GWAS are believed to represent the impact of genomic regulatory variants that cause modest transcriptional changes (GTExConsortium, 2015; Nicolae et al., 2010), it is intriguing that homologs of these genes are broadly expressed in *Drosophila*. Thus, future studies in flies could reveal the existence of conserved gene regulation and/or dose-sensitive requirements, consistent with the implicated mechanisms of susceptibility variants in human disease. Based on our results, however, we urge caution when drawing conclusions about cross-species functional conservation based solely on tissue expression patterns. Before deciding on the potential feasibility of pursuing follow-up studies in flies, it will be essential to integrate observations of expression with the results from directed experimental manipulations that yield more reliable evidence of conserved functional requirements, particularly within specific tissue contexts.

### Cross-species functional comparisons

Documenting candidate susceptibility gene conservation and expression in relevant tissues might be important foundations for follow-up of GWAS; however, the ultimate goal is to deploy powerful genetic models, including flies, for functional dissection. FlyBase provides an excellent starting point by not only documenting existing strains for experimental manipulation, but also making accessible the data on previously reported phenotypes (dos Santos et al., 2015). Quick lookups can often provide clues to the evolutionary conservation of functions, including those that are of potential relevance to human disease traits (Millburn et al., 2016). To illustrate this, we examined promising results from two selected GWAS that interrogated risk factors for social communication impairment in children, a trait related to autism spectrum disorders (St Pourcain et al., 2014, 2013). Together, these studies identified seven independent signals, of which five are conserved in *Drosophila*. Using FlyBase as a guide, along with targeted literature mining, we discovered that the reported loss-of-function phenotypes suggest conserved gene functions that might be relevant to their associations with the complex human phenotype of social communication. For example, *bru3*, the fly ortholog of human *CELF4*, encoding an RNA-binding protein and regulator of transcript splicing and translation, was identified in a *Drosophila* genetic screen for gender-specific social responsiveness (Ellis and Carney, 2011). Another gene, *Tmhs*, homologous to *LHFPL3*, encoding a tetraspan membrane protein, is required for fly auditory perception (Cosetti et al., 2008; Coop et al., 2008). Lastly, *CG4328*, a homolog of *LMX1B*, encoding a transcription factor, was linked to sensory neuron dendritic arborization (Parrish et al., 2006). Though speculative in the absence of further experimental validation, these published loss-of-function phenotypes in *Drosophila* suggest that the homologous human susceptibility genes might subserve similar functions in the genesis of social communication disorders. Thus, targeted data mining allows results of human GWAS to be rapidly linked to relevant model organism phenotypes, informing mechanistic hypotheses for further testing.

These examples provide potential support for the functional conservation of genes underlying complex human traits; nonetheless, it is difficult to generalize based on anecdotal evidence alone. In order to test the hypothesis more systematically, we again considered all human candidate susceptibility genes for neurological traits, asking whether conserved *Drosophila* homologs are similarly associated with nervous system phenotypes in flies. For this analysis, we took advantage of the FlyBase ‘controlled vocabulary (CV)’ terms used to standardize phenotype reporting (Millburn et al., 2016). Of the homologs for human neurological susceptibility genes, 33% (306 out of 914) are established to cause neuronal or nervous system phenotypes in *Drosophila*, based on 41 FlyBase CV terms, compared with 25% for all fly genes. Although this represents only a modest enrichment over the 29% of all GWAS candidate homologs causing fly neuronal phenotypes, it is potentially consistent with our finding (Fig. 4A), that such genes are frequently expressed in the *Drosophila* nervous system.

Although the neuronal functions of *Drosophila* genes are more widely annotated than in humans or mammalian models, one important caveat is that many genes still remain incompletely studied. Even for genes with well-characterized loss-of-function phenotypes, semantic barriers can complicate the precise matching of fly to human traits, because *Drosophila* and human geneticists frequently use widely differing terminologies to describe phenotypes (Pospisilik et al., 2010). Given the growing recognition of this challenge, phenotype curation in both humans and model organisms and associated bioinformatic tools for data integration are likely to improve in the near future (Deans et al., 2015; Mungall et al., 2010, 2015). Community resources, such as the Monarch Initiative (https://monarchinitiative.org), promise to facilitate mapping of human to model organism traits, thereby enhancing the functional follow-up of implicated susceptibility genes.

In sum, as our systematic understanding of the functional requirements of all conserved *Drosophila* genes improves, so will the analytical power to pinpoint human traits amenable to cross-species functional dissection. However, this conclusion depends on the likely flawed assumption that conserved genes and associated genetic pathways will cause similar loss-of-function phenotypes in distantly related organisms, such as humans and *Drosophila*. In fact, many strongly conserved molecular systems, such as signal transduction pathways or gene coexpression networks, are ‘repurposed’ over evolutionary timescales for heterogeneous cellular, developmental and organismal functions. For example, mutations in the human sonic hedgehog gene (*SHH*) result in nervous system pattering defects and subsequent developmental malformations (e.g. holoprosencephaly). By contrast, mutations in the *Drosophila* ortholog, *hedgehog* (*hh*), disrupt embryonic segmentation and lead to altered appearance of larval denticle belts. Nevertheless, studies of this larval phenotype have facilitated successful identification and mechanistic dissection of *hh* and numerous genes encoding highly conserved downstream signaling components. This and numerous similar examples illustrate that the mere superficial equivalence of *distal* phenotypic outcomes is likely a poor predictor for the potential value of flies in the mechanistic follow-up of human complex traits; rather, it is the fundamental conservation and coherence of the more *proximal* genetic regulatory networks that are consequential. To put this more simply, many potentially valuable *Drosophila* ‘disease models’ might have little or no resemblance to their cognate human traits. Embracing this insight, Marcotte and colleagues (McGary et al., 2010; Woods et al., 2013) deployed an unbiased bioinformatic approach to define homologous phenotypes, or ‘phenologs’ (http://www.phenologs.org), between distantly related species, including humans and several experimental animal models (but unfortunately not *Drosophila*). Phenolog assignment was based on overlap between groups of conserved gene sets that cause similar phenotypes within each species. For example, this successful strategy led to testable predictions of new genes involved in human breast cancer and neural crest defects based on non-obvious homologous phenotypes in *C. elegans* (hermaphroditism) and *Arabidopsis* (gravitropism), respectively (McGary et al., 2010).

Full implementation of the phenolog strategy in *Drosophila* is outside the scope of this Review; however, we did examine the proportion of fly homologs of human susceptibility gene candidates from GWAS that are essential (Table 3; full results in Table S4), i.e. result in embryonic lethality when genetically disrupted. As a group, human GWAS identify orthologous fly genes that are strongly enriched for lethal phenotypes (43% versus 25% for all *Drosophila* genes). Interestingly, among disease mechanism categories, candidate susceptibility genes for human developmental disorders were even more likely to be required for embryonic viability in flies (50% lethal phenotypes). More broadly, 66% of all homologs for human susceptibility gene candidates from GWAS currently have phenotypic annotations in FlyBase, providing an immediate entry point for functional study in flies, such as rapid tests of phenotypic rescue by the human homologs and/or evaluation of genetic interactions with other candidate susceptibility genes.

**Table 3.**
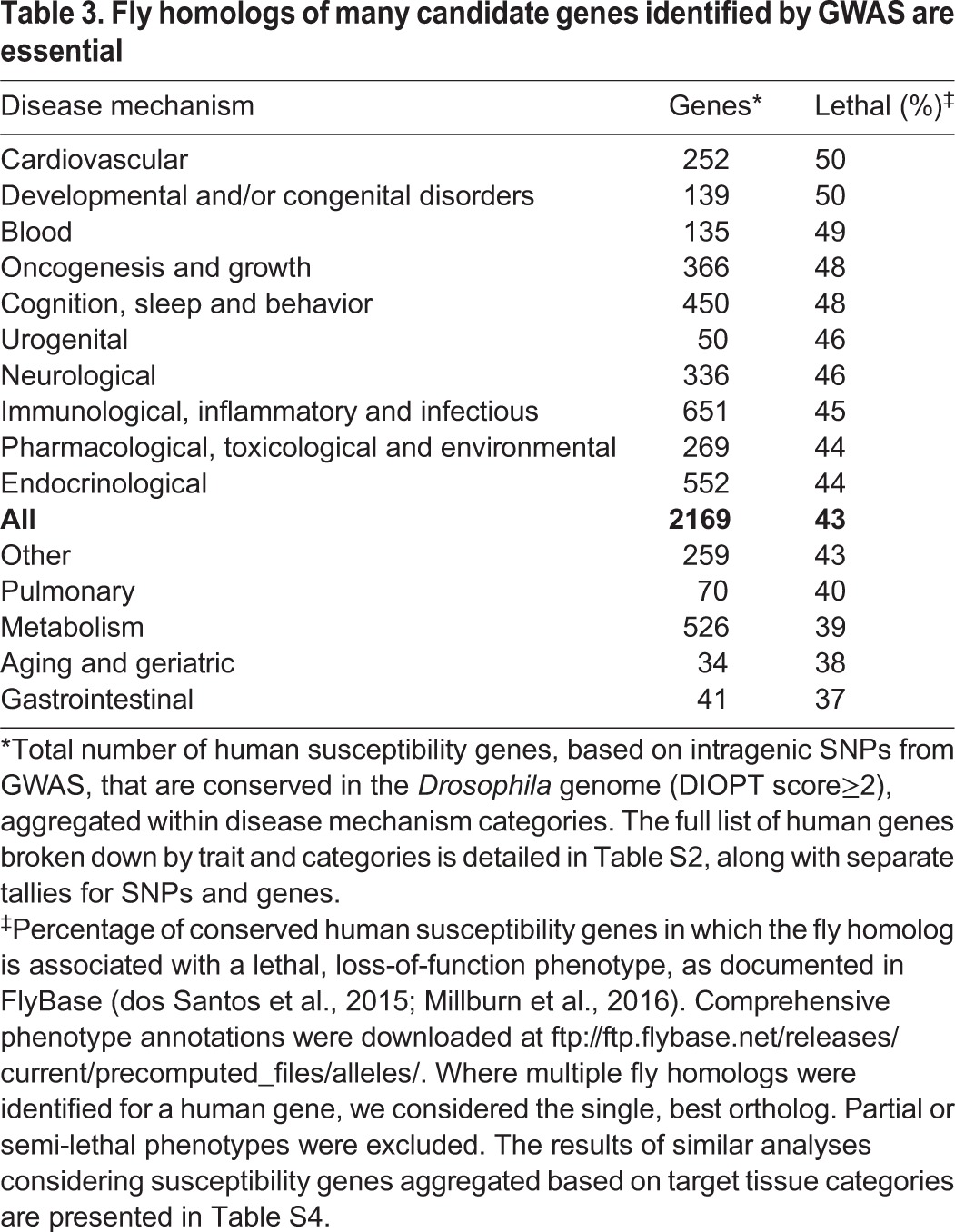
**Fly homologs of many candidate genes identified by GWAS are essential**

## Conclusions

Recently, the pre-eminence of GWAS as a tool for susceptibility locus discovery in human complex genetic disorders is being supplanted by next-generation sequencing approaches and the complementary search for rare variant risk alleles. This comes as the GWAS approach reaches maturity, with many common diseases having now been interrogated by meta-analyses that involve the largest feasible sample sizes. Similar to the arrival of GWAS about 10 years ago, this shift is further fueled by the tumbling cost of next-generation sequencing technology coupled with advances in statistical and analytical methods. Whereas GWAS usually implicates tag SNPs and genomic loci encompassing multiple potential causal genes, sequencing instead promises to pinpoint specific gene variants with putative functional consequences. Nevertheless, the large number of implicated variants emerging from most sequencing studies leads to a related set of challenges as well as opportunities for experimental follow-up (Shulman, 2015). While embracing the opportunities for sequencing-based discovery, it is essential that we not prematurely abandon GWAS, given the potential of these data to illuminate the yet largely unknown biological mechanisms underlying common diseases with complex genetic etiologies. Indeed, as we have emphasized throughout this Review, although the human genetic work might be winding down, a significant challenge remains to confirm the responsible genes and understand the relevant mechanisms, a task well-suited for the model organism research community.

Although we have focused on the powerful tools and approaches that *Drosophila* models can bring to bear on GWAS follow-up, other experimental systems also have important roles to play. Integrated studies in both simple (e.g. yeast, nematode, fly) and more complex (e.g. zebrafish, mouse) animal models can create powerful synergy for the follow-up of candidates arising from human genetic studies, highlighting the evolutionary conservation of disease mechanisms. Notably, recent advances in human induced pluripotent stem cell (iPSC) methods offer powerful, complementary experimental systems for functional validation of GWAS findings (Zhu et al., 2011). Further, the application of high-throughput technologies for profiling the epigenome, transcriptome, proteome, and/or metabolome in human tissues, coupled with robust systems biology approaches, show increasing promise for mechanistic dissection. In fact, some have argued that such advances could render model organisms obsolete for the study of complex human traits (Visscher, 2016). In our opinion, such predictions represent a grave misreading of the current research landscape. Despite offering the distinct advantage of a human genomic context for variant validation and more faithful recapitulation of the species-specific cellular milieu, iPSC-based approaches nevertheless fall short in several important areas. The mechanisms underlying complex human traits can rarely be reduced to single cells but rather play out at the tissue or even multi-system level, requiring *in vivo*, organismal models for mechanistic dissection. Other important factors contributing to complex traits that are challenging, if not impossible, to approximate in cell culture include the impact of developmental biology and aging. Moreover, bioinformatics using large-scale, comprehensive ’omic data can be extraordinarily powerful for hypothesis generation. However, results from these analyses are usually correlative in nature, crucially requiring experimental validation to establish causation (Chakravarti et al., 2013). All experimental systems, including both *Drosophila* and human subject investigations, have their limitations. As alluded to earlier, many, but not all, genes are conserved, and there are certainly many facets of human disease that are likely not amenable to modeling in *Drosophila*. Nevertheless, our analyses demonstrate how a majority of human susceptibility loci are highly conserved in flies, creating myriad opportunities for functional follow-up, even where such genes might be operating in different contexts. Given the remarkable scope of the challenges currently encountered in functional genomics, we must leverage all available tools to address these important problems, and it is incumbent upon all stakeholders to embrace such efforts, including investigators, funding bodies and publishers.

In sum, understanding the mechanisms of susceptibility for common and complex genetic diseases is an urgent public health priority. These disorders – including heart/lung disease, cancer, stroke, Alzheimer's disease, diabetes, and many others – account for the overwhelming population burden of morbidity and mortality in the developed world. The successful identification of risk loci by GWAS provides an enormous opportunity for translational research aimed at discovering completely novel drug targets. As highlighted here, susceptibility locus discovery is only the first step, with the crucial challenge remaining to define the relevant mechanisms. Only then can the therapeutic potential of GWAS findings be fully unleashed. We hope that this Review makes a compelling case that *Drosophila* models offer one important path forward.

## References

[DMM027680C1] Balding D. J. (2006). A tutorial on statistical methods for population association studies. *Nat. Rev. Genet.* 7, 781-791. 10.1038/nrg1916 16983374

[DMM027680C2] Bellen H. J. and Yamamoto S. (2015). Morgan's legacy: fruit flies and the functional annotation of conserved genes. *Cell* 163, 12-14. 10.1016/j.cell.2015.09.009 26406362 PMC4783153

[DMM027680C3] Bellen H. J., Levis R. W., He Y., Carlson J. W., Evans-Holm M., Bae E., Kim J., Metaxakis A., Savakis C., Schulze K. L. et al. (2011). The Drosophila gene disruption project: progress using transposons with distinctive site specificities. *Genetics* 188, 731-743. 10.1534/genetics.111.126995 21515576 PMC3176542

[DMM027680C4] Bonnefond A. and Froguel P. (2015). Rare and common genetic events in type 2 diabetes: what should biologists know? *Cell Metab.* 21, 357-368. 10.1016/j.cmet.2014.12.020 25640731

[DMM027680C5] Brand A. and Perrimon N. (1993). Targeted gene expression as a means of altering cell fates and generating dominant phenotypes. *Development* 118, 401-415. 8223268 10.1242/dev.118.2.401

[DMM027680C6] Brown J. B., Boley N., Eisman R., May G. E., Stoiber M. H., Duff M. O., Booth B. W., Wen J., Park S., Suzuki A. M. et al. (2014). Diversity and dynamics of the Drosophila transcriptome. *Nature* 512, 393-399. 10.1038/nature12962 24670639 PMC4152413

[DMM027680C7] Cantor R. M., Lange K. and Sinsheimer J. S. (2010). Prioritizing GWAS results: A review of statistical methods and recommendations for their application. *Am. J. Hum. Genet.* 86, 6-22. 10.1016/j.ajhg.2009.11.017 20074509 PMC2801749

[DMM027680C8] Chakravarti A., Clark A. G. and Mootha V. K. (2013). Distilling pathophysiology from complex disease genetics. *Cell* 155, 21-26. 10.1016/j.cell.2013.09.001 24074858 PMC4244836

[DMM027680C9] Chapuis J., Hansmannel F., Gistelinck M., Mounier A., Van Cauwenberghe C., Kolen K. V., Geller F., Sottejeau Y., Harold D., Dourlen P. et al. (2013). Increased expression of BIN1 mediates Alzheimer genetic risk by modulating tau pathology. *Mol. Psychiatry* 18, 1225-1234. 10.1038/mp.2013.1 23399914 PMC3807661

[DMM027680C10] Cook R. K., Christensen S. J., Deal J. A., Coburn R. A., Deal M. E., Gresens J. M., Kaufman T. C. and Cook K. R. (2012). The generation of chromosomal deletions to provide extensive coverage and subdivision of the Drosophila melanogaster genome. *Genome Biol.* 13, R21 10.1186/gb-2012-13-3-r21 22445104 PMC3439972

[DMM027680C11] Coop G., Wen X., Ober C., Pritchard J. K. and Przeworski M. (2008). High-resolution mapping of crossovers reveals extensive variation in fine-scale recombination patterns among humans. *Science* 319, 1395-1398. 10.1126/science.1151851 18239090

[DMM027680C12] Corder E. H., Saunders A. M., Strittmatter W. J., Schmechel D. E., Gaskell P. C., Small G. W., Roses A. D., Haines J. L. and Pericak-Vance M. A. (1993). Gene dose of apolipoprotein E type 4 allele and the risk of Alzheimer's disease in late onset families. *Science* 261, 921-923. 10.1126/science.8346443 8346443

[DMM027680C13] Cosetti M., Culang D., Kotla S., O'Brien P., Eberl D. F. and Hannan F. (2008). Unique transgenic animal model for hereditary hearing loss. *Ann. Otol. Rhinol. Laryngol.* 117, 827-833. 10.1177/000348940811701106 19102128 PMC3409696

[DMM027680C14] De Jager P. L., Jia X., Wang J., de Bakker P. I. W., Ottoboni L., Aggarwal N. T., Piccio L., Raychaudhuri S., Tran D., Aubin C. et al. (2009). Meta-analysis of genome scans and replication identify CD6, IRF8 and TNFRSF1A as new multiple sclerosis susceptibility loci. *Nat. Genet.* 41, 776-782. 10.1038/ng.401 19525953 PMC2757648

[DMM027680C15] Deans A. R., Lewis S. E., Huala E., Anzaldo S. S., Ashburner M., Balhoff J. P., Blackburn D. C., Blake J. A., Burleigh J. G., Chanet B. et al. (2015). Finding our way through phenotypes. *PLoS Biol.* 13, e1002033 10.1371/journal.pbio.1002033 25562316 PMC4285398

[DMM027680C16] Dembeck L. M., Huang W., Magwire M. M., Lawrence F., Lyman R. F. and Mackay T. F. C. (2015). Genetic architecture of abdominal pigmentation in Drosophila melanogaster. *PLoS Genet.* 11, e1005163 10.1371/journal.pgen.1005163 25933381 PMC4416719

[DMM027680C17] Dietzl G., Chen D., Schnorrer F., Su K.-C., Barinova Y., Fellner M., Gasser B., Kinsey K., Oppel S., Scheiblauer S. et al. (2007). A genome-wide transgenic RNAi library for conditional gene inactivation in Drosophila. *Nature* 448, 151-156. 10.1038/nature05954 17625558

[DMM027680C18] Diop S. B., Bisharat-Kernizan J., Birse R. T., Oldham S., Ocorr K. and Bodmer R. (2015). PGC-1/spargel counteracts high-fat-diet-induced obesity and cardiac lipotoxicity downstream of TOR and brummer ATGL lipase. *Cell Rep.* 10, 1572-1584. 10.1016/j.celrep.2015.02.022 PMC4560688 25753422

[DMM027680C19] dos Santos G., Schroeder A. J., Goodman J. L., Strelets V. B., Crosby M. A., Thurmond J., Emmert D. B. and Gelbart W. M. (2015). FlyBase: introduction of the Drosophila melanogaster Release 6 reference genome assembly and large-scale migration of genome annotations. *Nucleic Acids Res.* 43, D690-D697. 10.1093/nar/gku1099 25398896 PMC4383921

[DMM027680C20] Edwards S. L., Beesley J., French J. D. and Dunning A. M. (2013). Beyond GWASs: illuminating the dark road from association to function. *Am. J. Hum. Genet.* 93, 779-797. 10.1016/j.ajhg.2013.10.012 24210251 PMC3824120

[DMM027680C21] Ellis L. L. and Carney G. E. (2011). Socially-responsive gene expression in male Drosophila melanogaster is influenced by the sex of the interacting partner. *Genetics* 187, 157-169. 10.1534/genetics.110.122754 20980240 PMC3018301

[DMM027680C22] EncodeProjectConsortium (2004). The ENCODE (ENCyclopedia Of DNA Elements) Project. *Science* 306, 636-640. 10.1126/science.1105136 15499007

[DMM027680C23] Fortini M. E., Skupski M. P., Boguski M. S. and Hariharan I. K. (2000). A survey of human disease gene counterparts in the Drosophila genome. *J. Cell Biol.* 150, F23-F30. 10.1083/jcb.150.2.F23 10908582 PMC2180233

[DMM027680C24] Francioli L. C., Menelaou A., Pulit S. L., van Dijk F., Palamara P. F., Elbers C., Neerincx P. B. T., Ye K., Guryev V., Kloosterman W. P. et al. (2014). Whole-genome sequence variation, population structure and demographic history of the Dutch population. *Nat. Genet.* 46, 818-825. 10.1038/ng.3021 24974849

[DMM027680C25] Frazer K. A. Ballinger D. G. Cox D. R. Hinds D. A. Stuve L. L. Gibbs R. A. Belmont J. W. Boudreau A. Hardenbol P. Leal S. M. et al. (2007). A second generation human haplotype map of over 3.1 million SNPs. *Nature* 449, 851-861. 10.1038/nature06258 17943122 PMC2689609

[DMM027680C26] Freeman A., Pranski E., Miller R. D., Radmard S., Bernhard D., Jinnah H. A., Betarbet R., Rye D. B. and Sanyal S. (2012). Sleep fragmentation and motor restlessness in a Drosophila model of Restless Legs Syndrome. *Curr. Biol.* 22, 1142-1148. 10.1016/j.cub.2012.04.027 22658601 PMC3381864

[DMM027680C27] Gaertner B. E., Ruedi E. A., McCoy L. J., Moore J. M., Wolfner M. F. and Mackay T. F. C. (2015). Heritable variation in courtship patterns in Drosophila melanogaster. *G3* 5, 531-539. 10.1534/g3.114.014811 25650358 PMC4390569

[DMM027680C28] Goldstein D. B., Allen A., Keebler J., Margulies E. H., Petrou S., Petrovski S. and Sunyaev S. (2013). Sequencing studies in human genetics: design and interpretation. *Nat. Rev. Genet.* 14, 460-470. 10.1038/nrg3455 23752795 PMC4117319

[DMM027680C29] GTExConsortium (2015). Human genomics. The Genotype-Tissue Expression (GTEx) pilot analysis: multitissue gene regulation in humans. *Science* 348, 648-660. 10.1126/science.1262110 25954001 PMC4547484

[DMM027680C30] Gudbjartsson D. F., Helgason H., Gudjonsson S. A., Zink F., Oddson A., Gylfason A., Besenbacher S., Magnusson G., Halldorsson B. V., Hjartarson E. et al. (2015). Large-scale whole-genome sequencing of the Icelandic population. *Nat. Genet.* 47, 435-444. 10.1038/ng.3247 25807286

[DMM027680C31] Haelterman N. A., Yoon W. H., Sandoval H., Jaiswal M., Shulman J. M. and Bellen H. J. (2014). A mitocentric view of Parkinson's disease. *Annu. Rev. Neurosci.* 37, 137-159. 10.1146/annurev-neuro-071013-014317 24821430 PMC4659514

[DMM027680C32] Harbison S. T., McCoy L. J. and Mackay T. F. C. (2013). Genome-wide association study of sleep in Drosophila melanogaster. *BMC Genomics* 14, 281 10.1186/1471-2164-14-281 23617951 PMC3644253

[DMM027680C33] Hardy J. and Singleton A. (2009). Genomewide association studies and human disease. *N. Engl. J. Med.* 360, 1759-1768. 10.1056/NEJMra0808700 19369657 PMC3422859

[DMM027680C34] Hindorff L. A., Sethupathy P., Junkins H. A., Ramos E. M., Mehta J. P., Collins F. S. and Manolio T. A. (2009). Potential etiologic and functional implications of genome-wide association loci for human diseases and traits. *Proc. Natl. Acad. Sci. USA* 106, 9362-9367. 10.1073/pnas.0903103106 19474294 PMC2687147

[DMM027680C35] Hu Y., Flockhart I., Vinayagam A., Bergwitz C., Berger B., Perrimon N. and Mohr S. E. (2011). An integrative approach to ortholog prediction for disease-focused and other functional studies. *BMC Bioinformatics* 12, 357 10.1186/1471-2105-12-357 21880147 PMC3179972

[DMM027680C36] Hu Z., Li Z., Yu J., Tong C., Lin Y., Guo X., Lu F., Dong J., Xia Y., Wen Y. et al. (2014). Association analysis identifies new risk loci for non-obstructive azoospermia in Chinese men. *Nat. Commun.* 5, 3857 10.1038/ncomms4857 24852083

[DMM027680C37] Ioannidis J. P. A., Thomas G. and Daly M. J. (2009). Validating, augmenting and refining genome-wide association signals. *Nat. Rev. Genet.* 10, 318-329. 10.1038/nrg2544 19373277 PMC7877552

[DMM027680C38] Ivanov D. K., Escott-Price V., Ziehm M., Magwire M. M., Mackay T. F. C., Partridge L. and Thornton J. M. (2015). Longevity GWAS using the Drosophila genetic reference panel. *J. Gerontol. A Biol. Sci. Med. Sci.* 70, 1470-1478. 10.1093/gerona/glv047 25922346 PMC4631106

[DMM027680C39] Ivatt R. M., Sanchez-Martinez A., Godena V. K., Brown S., Ziviani E. and Whitworth A. J. (2014). Genome-wide RNAi screen identifies the Parkinson disease GWAS risk locus SREBF1 as a regulator of mitophagy. *Proc. Natl. Acad. Sci. USA* 111, 8494-8499. 10.1073/pnas.1321207111 24912190 PMC4060696

[DMM027680C40] Jaiswal M., Sandoval H., Zhang K., Bayat V. and Bellen H. J. (2012). Probing mechanisms that underlie human neurodegenerative diseases in Drosophila. *Annu. Rev. Genet.* 46, 371-396. 10.1146/annurev-genet-110711-155456 22974305 PMC3663445

[DMM027680C41] Lambert J.-C., Ibrahim-Verbaas C. A., Harold D., Naj A. C., Sims R., Bellenguez C., DeStafano A. L., Bis J. C., Beecham G. W., Grenier-Boley B. et al. (2013). Meta-analysis of 74,046 individuals identifies 11 new susceptibility loci for Alzheimer's disease. *Nat. Genet.* 45, 1452-1458. 10.1038/ng.2802 24162737 PMC3896259

[DMM027680C42] Lee J. M., Wheeler V. C., Chao M. J., Vonsattel J. P., Pinto R. M., Lucente D., Abu-Elneel K., Ramos E. M., Mysore J. S., Gillis T. et al. (2015). Identification of genetic factors that modify clinical onset of Huntington's disease. *Cell* 162, 516-526. 10.1016/j.cell.2015.07.003 26232222 PMC4524551

[DMM027680C43] Lessing D. and Bonini N. M. (2009). Maintaining the brain: insight into human neurodegeneration from Drosophila melanogaster mutants. *Nat. Rev. Genet.* 10, 359-370. 10.1038/nrg2563 19434080 PMC2820605

[DMM027680C44] Lu X., Wang L., Chen S., He L., Yang X., Shi Y., Cheng J., Zhang L., Gu C. C., Huang J. et al. (2012). Genome-wide association study in Han Chinese identifies four new susceptibility loci for coronary artery disease. *Nat. Genet.* 44, 890-894. 10.1038/ng.2337 22751097 PMC3927410

[DMM027680C45] MacLeod D. A., Rhinn H., Kuwahara T., Zolin A., Di Paolo G., McCabe B. D., Marder K. S., Honig L. S., Clark L. N., Small S. A. et al. (2013). RAB7L1 interacts with LRRK2 to modify intraneuronal protein sorting and Parkinson's disease risk. *Neuron* 77, 425-439. 10.1016/j.neuron.2012.11.033 23395371 PMC3646583

[DMM027680C46] Manolio T. A. (2013). Bringing genome-wide association findings into clinical use. *Nat. Rev. Genet.* 14, 549-558. 10.1038/nrg3523 23835440

[DMM027680C48] Matthews K. A., Kaufman T. C. and Gelbart W. M. (2005). Research resources for Drosophila: the expanding universe. *Nat. Rev. Genet.* 6, 179-193. 10.1038/nrg1554 15738962

[DMM027680C49] McGary K. L., Park T. J., Woods J. O., Cha H. J., Wallingford J. B. and Marcotte E. M. (2010). Systematic discovery of nonobvious human disease models through orthologous phenotypes. *Proc. Natl. Acad. Sci. USA* 107, 6544-6549. 10.1073/pnas.0910200107 20308572 PMC2851946

[DMM027680C50] Millburn G. H., Crosby M. A., Gramates L. S. and Tweedie S. and the FlyBase Consortium (2016). FlyBase portals to human disease research using Drosophila models. *Dis. Model. Mech.* 9, 245-252. 10.1242/dmm.023317 26935103 PMC4826978

[DMM027680C51] Mohr S. E., Hu Y., Kim K., Housden B. E. and Perrimon N. (2014). Resources for functional genomics studies in Drosophila melanogaster. *Genetics* 197, 1-18. 10.1534/genetics.113.154344 24653003 PMC4012471

[DMM027680C52] Moreau K., Fleming A., Imarisio S., Lopez Ramirez A., Mercer J. L., Jimenez-Sanchez M., Bento C. F., Puri C., Zavodszky E., Siddiqi F. et al. (2014). PICALM modulates autophagy activity and tau accumulation. *Nat. Commun.* 5, 4998 10.1038/ncomms5998 25241929 PMC4199285

[DMM027680C53] Mungall C. J., Gkoutos G. V., Smith C. L., Haendel M. A., Lewis S. E. and Ashburner M. (2010). Integrating phenotype ontologies across multiple species. *Genome Biol.* 11, R2 10.1186/gb-2010-11-1-r2 20064205 PMC2847714

[DMM027680C54] Mungall C. J., Washington N. L., Nguyen-Xuan J., Condit C., Smedley D., Köhler S., Groza T., Shefchek K., Hochheiser H., Robinson P. N. et al. (2015). Use of model organism and disease databases to support matchmaking for human disease gene discovery. *Hum. Mutat.* 36, 979-984. 10.1002/humu.22857 26269093 PMC5473253

[DMM027680C55] Naj A. C., Jun G., Beecham G. W., Wang L.-S., Vardarajan B. N., Buros J., Gallins P. J., Buxbaum J. D., Jarvik G. P., Crane P. K. et al. (2011). Common variants at MS4A4/MS4A6E, CD2AP, CD33 and EPHA1 are associated with late-onset Alzheimer's disease. *Nat. Genet.* 43, 436-441. 10.1038/ng.801 21460841 PMC3090745

[DMM027680C56] Nalls M. A., Pankratz N., Lill C. M., Do C. B., Hernandez D. G., Saad M., DeStefano A. L., Kara E., Bras J., Sharma M. et al. (2014). Large-scale meta-analysis of genome-wide association data identifies six new risk loci for Parkinson's disease. *Nat. Genet.* 46, 989-993. 10.1038/ng.3043 25064009 PMC4146673

[DMM027680C57] Neely G. G., Hess A., Costigan M., Keene A. C., Goulas S., Langeslag M., Griffin R. S., Belfer I., Dai F., Smith S. B. et al. (2010a). A genome-wide Drosophila screen for heat nociception identifies alpha2delta3 as an evolutionarily conserved pain gene. *Cell* 143, 628-638. 10.1016/j.cell.2010.09.047 21074052 PMC3040441

[DMM027680C58] Neely G. G., Kuba K., Cammarato A., Isobe K., Amann S., Zhang L., Murata M., Elmén L., Gupta V., Arora S. et al. (2010b). A global in vivo Drosophila RNAi screen identifies NOT3 as a conserved regulator of heart function. *Cell* 141, 142-153. 10.1016/j.cell.2010.02.023 20371351 PMC2855221

[DMM027680C59] Nicolae D. L., Gamazon E., Zhang W., Duan S., Dolan M. E. and Cox N. J. (2010). Trait-associated SNPs are more likely to be eQTLs: annotation to enhance discovery from GWAS. *PLoS Genet.* 6, e1000888 10.1371/journal.pgen.1000888 20369019 PMC2848547

[DMM027680C60] Parrish J. Z., Kim M. D., Jan L. Y. and Jan Y. N. (2006). Genome-wide analyses identify transcription factors required for proper morphogenesis of Drosophila sensory neuron dendrites. *Genes Dev.* 20, 820-835. 10.1101/gad.1391006 16547170 PMC1472285

[DMM027680C61] Pendse J., Ramachandran P. V., Na J., Narisu N., Fink J. L., Cagan R. L., Collins F. S. and Baranski T. J. (2013). A Drosophila functional evaluation of candidates from human genome-wide association studies of type 2 diabetes and related metabolic traits identifies tissue-specific roles for dHHEX. *BMC Genomics* 14, 136 10.1186/1471-2164-14-136 23445342 PMC3608171

[DMM027680C62] Pericak-Vance M. A., Bebout J. L., Gaskell P. C. Jr., Yamaoka L. H., Hung W. Y., Alberts M. J., Walker A. P., Bartlett R. J., Haynes C. A., Welsh K. A. et al. (1991). Linkage studies in familial Alzheimer disease: evidence for chromosome 19 linkage. *Am. J. Hum. Genet.* 48, 1034-1050. 2035524 PMC1683100

[DMM027680C63] Perkins L. A., Holderbaum L., Tao R., Hu Y., Sopko R., McCall K., Yang-Zhou D., Flockhart I., Binari R., Shim H.-S. et al. (2015). The transgenic RNAi project at harvard medical school: resources and validation. *Genetics* 201, 843-852. 10.1534/genetics.115.180208 26320097 PMC4649654

[DMM027680C64] Pospisilik J. A., Schramek D., Schnidar H., Cronin S. J. F., Nehme N. T., Zhang X., Knauf C., Cani P. D., Aumayr K., Todoric J. et al. (2010). Drosophila genome-wide obesity screen reveals hedgehog as a determinant of brown versus white adipose cell fate. *Cell* 140, 148-160. 10.1016/j.cell.2009.12.027 20074523

[DMM027680C65] Ripke S., O'Dushlaine C., Chambert K., Moran J. L., Kähler A. K., Akterin S., Bergen S. E., Collins A. L., Crowley J. J., Fromer M. et al. (2013). Genome-wide association analysis identifies 13 new risk loci for schizophrenia. *Nat. Genet.* 45, 1150-1159. 10.1038/ng.2742 23974872 PMC3827979

[DMM027680C66] Romero E., Cha G.-H., Verstreken P., Ly C. V., Hughes R. E., Bellen H. J. and Botas J. (2008). Suppression of neurodegeneration and increased neurotransmission caused by expanded full-length huntingtin accumulating in the cytoplasm. *Neuron* 57, 27-40. 10.1016/j.neuron.2007.11.025 18184562 PMC2277511

[DMM027680C67] Ryder E., Ashburner M., Bautista-Llacer R., Drummond J., Webster J., Johnson G., Morley T., Chan Y. S., Blows F., Coulson D. et al. (2007). The DrosDel deletion collection: a Drosophila genomewide chromosomal deficiency resource. *Genetics* 177, 615-629. 10.1534/genetics.107.076216 17720900 PMC2013729

[DMM027680C68] Sawcer S., Hellenthal G., Pirinen M., Spencer C. C., Patsopoulos N. A., Moutsianas L., Dilthey A., Su Z., Freeman C., Hunt S. E. et al. (2011). Genetic risk and a primary role for cell-mediated immune mechanisms in multiple sclerosis. *Nature* 476, 214-219. 10.1038/nature10251 21833088 PMC3182531

[DMM027680C69] Schumann G., Coin L. J., Lourdusamy A., Charoen P., Berger K. H., Stacey D., Desrivieres S., Aliev F. A., Khan A. A., Amin N. et al. (2011). Genome-wide association and genetic functional studies identify autism susceptibility candidate 2 gene (AUTS2) in the regulation of alcohol consumption. *Proc. Natl. Acad. Sci. USA* 108, 7119-7124. 10.1073/pnas.1017288108 21471458 PMC3084048

[DMM027680C70] Schunkert H., König I. R., Kathiresan S., Reilly M. P., Assimes T. L., Holm H., Preuss M., Stewart A. F. R., Barbalic M., Gieger C. et al. (2011). Large-scale association analysis identifies 13 new susceptibility loci for coronary artery disease. *Nat. Genet.* 43, 333-338. 10.1038/ng.784 21378990 PMC3119261

[DMM027680C71] Shi Y., Li Z., Xu Q., Wang T., Li T., Shen J., Zhang F., Chen J., Zhou G., Ji W. et al. (2011). Common variants on 8p12 and 1q24.2 confer risk of schizophrenia. *Nat. Genet.* 43, 1224-1227. 10.1038/ng.980 22037555 PMC3773910

[DMM027680C72] Shorter J., Couch C., Huang W., Carbone M. A., Peiffer J., Anholt R. R. H. and Mackay T. F. C. (2015). Genetic architecture of natural variation in Drosophila melanogaster aggressive behavior. *Proc. Natl. Acad. Sci. USA* 112, E3555-E3563. 10.1073/pnas.1510104112 26100892 PMC4500262

[DMM027680C73] Shulman J. M. (2015). Drosophila and experimental neurology in the post-genomic era. *Exp. Neurol.* 274, 4-13. 10.1016/j.expneurol.2015.03.016 25814441 PMC4962325

[DMM027680C74] Shulman J. M., Shulman L. M., Weiner W. J. and Feany M. B. (2003). From fruit fly to bedside: translating lessons from Drosophila models of neurodegenerative disease. *Curr. Opin. Neurol.* 16, 443-449. 10.1097/01.wco.0000084220.82329.60 12869801

[DMM027680C75] Shulman J. M., Chipendo P., Chibnik L. B., Aubin C., Tran D., Keenan B. T., Kramer P. L., Schneider J. A., Bennett D. A., Feany M. B. et al. (2011). Functional screening of Alzheimer pathology genome-wide association signals in Drosophila. *Am. J. Hum. Genet.* 88, 232-238. 10.1016/j.ajhg.2011.01.006 21295279 PMC3035702

[DMM027680C76] Shulman J. M., Imboywa S., Giagtzoglou N., Powers M. P., Hu Y., Devenport D., Chipendo P., Chibnik L. B., Diamond A., Perrimon N. et al. (2014). Functional screening in Drosophila identifies Alzheimer's disease susceptibility genes and implicates Tau-mediated mechanisms. *Hum. Mol. Genet.* 23, 870-877. 10.1093/hmg/ddt478 24067533 PMC3900103

[DMM027680C77] Smoller J., Ripke S., Lee P., Neale B. and Ji N. (2013). Identification of risk loci with shared effects on five major psychiatric disorders: a genome-wide analysis. *Lancet* 381, 1371-1379. 10.1016/S0140-6736(12)62129-1 23453885 PMC3714010

[DMM027680C78] Solovieff N., Cotsapas C., Lee P. H., Purcell S. M. and Smoller J. W. (2013). Pleiotropy in complex traits: challenges and strategies. *Nat. Rev. Genet.* 14, 483-495. 10.1038/nrg3461 23752797 PMC4104202

[DMM027680C79] St Pourcain B., Whitehouse A. J. O., Ang W. Q., Warrington N. M., Glessner J. T., Wang K., Timpson N. J., Evans D. M., Kemp J. P., Ring S. M. et al. (2013). Common variation contributes to the genetic architecture of social communication traits. *Mol. Autism* 4, 34 10.1186/2040-2392-4-34 24047820 PMC3853437

[DMM027680C80] St Pourcain B., Skuse D. H., Mandy W. P., Wang K., Hakonarson H., Timpson N. J., Evans D. M., Kemp J. P., Ring S. M., McArdle W. L. et al. (2014). Variability in the common genetic architecture of social-communication spectrum phenotypes during childhood and adolescence. *Mol. Autism* 5, 18 10.1186/2040-2392-5-18 24564958 PMC3940728

[DMM027680C81] Stefansson H., Rye D. B., Hicks A., Petursson H., Ingason A., Thorgeirsson T. E., Palsson S., Sigmundsson T., Sigurdsson A. P., Eiriksdottir I. et al. (2007). A genetic risk factor for periodic limb movements in sleep. *N. Engl. J. Med.* 357, 639-647. 10.1056/NEJMoa072743 17634447

[DMM027680C82] Takahara B. and Takahashi K. H. (2015). Genome-wide association study on male genital shape and size in Drosophila melanogaster. *PLoS ONE* 10, e0132846 10.1371/journal.pone.0132846 26182199 PMC4504508

[DMM027680C83] Teslovich T. M., Musunuru K., Smith A. V., Edmondson A. C., Stylianou I. M., Koseki M., Pirruccello J. P., Ripatti S., Chasman D. I., Willer C. J. et al. (2010). Biological, clinical and population relevance of 95 loci for blood lipids. *Nature* 466, 707-713. 10.1038/nature09270 20686565 PMC3039276

[DMM027680C84] Tsuda H., Jafar-Nejad H., Patel A. J., Sun Y., Chen H.-K., Rose M. F., Venken K. J. T., Botas J., Orr H. T., Bellen H. J. et al. (2005). The AXH domain of Ataxin-1 mediates neurodegeneration through its interaction with Gfi-1/Senseless proteins. *Cell* 122, 633-644. 10.1016/j.cell.2005.06.012 16122429

[DMM027680C85] Tsuda H., Han S. M., Yang Y., Tong C., Lin Y. Q., Mohan K., Haueter C., Zoghbi A., Harati Y., Kwan J. et al. (2008). The amyotrophic lateral sclerosis 8 protein VAPB is cleaved, secreted, and acts as a ligand for Eph receptors. *Cell* 133, 963-977. 10.1016/j.cell.2008.04.039 18555774 PMC2494862

[DMM027680C86] Ugur B., Chen K. and Bellen H. J. (2016). Drosophila tools and assays for the study of human diseases. *Dis. Model. Mech.* 9, 235-244. 10.1242/dmm.023762 26935102 PMC4833332

[DMM027680C87] Venkatachalam K., Long A. A., Elsaesser R., Nikolaeva D., Broadie K. and Montell C. (2008). Motor deficit in a Drosophila model of mucolipidosis type IV due to defective clearance of apoptotic cells. *Cell* 135, 838-851. 10.1016/j.cell.2008.09.041 19041749 PMC2649760

[DMM027680C88] Venken K. J. T., Simpson J. H. and Bellen H. J. (2011). Genetic manipulation of genes and cells in the nervous system of the fruit fly. *Neuron* 72, 202-230. 10.1016/j.neuron.2011.09.021 22017985 PMC3232021

[DMM027680C89] Visscher P. M. (2016). Human complex trait genetics in the 21st century. *Genetics* 202, 377-379. 10.1534/genetics.115.180513 26869482 PMC4788221

[DMM027680C90] Voght S. P., Fluegel M. L., Andrews L. A. and Pallanck L. J. (2007). Drosophila NPC1b promotes an early step in sterol absorption from the midgut epithelium. *Cell Metab.* 5, 195-205. 10.1016/j.cmet.2007.01.011 17339027

[DMM027680C91] Vos M., Esposito G., Edirisinghe J. N., Vilain S., Haddad D. M., Slabbaert J. R., Van Meensel S., Schaap O., De Strooper B., Meganathan R. et al. (2012). Vitamin K2 is a mitochondrial electron carrier that rescues pink1 deficiency. *Science* 336, 1306-1310. 10.1126/science.1218632 22582012

[DMM027680C92] Wall J. D. and Pritchard J. K. (2003). Assessing the performance of the haplotype block model of linkage disequilibrium. *Am. J. Hum. Genet.* 73, 502-515. 10.1086/378099 12916017 PMC1180676

[DMM027680C93] Walter K., Min J. L., Huang J., Crooks L., Memari Y., McCarthy S., Perry J. R. B., Xu C., Futema M., Lawson D. et al. (2015). The UK10K project identifies rare variants in health and disease. *Nature* 526, 82-90. 10.1038/nature14962 26367797 PMC4773891

[DMM027680C94] Wang S., Tan K. L., Agosto M. A., Xiong B., Yamamoto S., Sandoval H., Jaiswal M., Bayat V., Zhang K., Charng W.-L. et al. (2014). The retromer complex is required for rhodopsin recycling and its loss leads to photoreceptor degeneration. *PLoS Biol.* 12, e1001847 10.1371/journal.pbio.1001847 24781186 PMC4004542

[DMM027680C95] Wangler M. F., Yamamoto S. and Bellen H. J. (2015). Fruit flies in biomedical research. *Genetics* 199, 639-653. 10.1534/genetics.114.171785 25624315 PMC4349060

[DMM027680C96] Weavers H., Prieto-Sánchez S., Grawe F., Garcia-López A., Artero R., Wilsch-Bräuninger M., Ruiz-Gómez M., Skaer H. and Denholm B. (2009). The insect nephrocyte is a podocyte-like cell with a filtration slit diaphragm. *Nature* 457, 322-326. 10.1038/nature07526 18971929 PMC2687078

[DMM027680C97] Welter D., MacArthur J., Morales J., Burdett T., Hall P., Junkins H., Klemm A., Flicek P., Manolio T., Hindorff L. et al. (2014). The NHGRI GWAS Catalog, a curated resource of SNP-trait associations. *Nucleic Acids Res.* 42, D1001-D1006. 10.1093/nar/gkt1229 24316577 PMC3965119

[DMM027680C98] Woods J. O., Singh-Blom U. M., Laurent J. M., McGary K. L. and Marcotte E. M. (2013). Prediction of gene–phenotype associations in humans, mice, and plants using phenologs. *BMC Bioinformatics* 14, 203 10.1186/1471-2105-14-203 23800157 PMC3704650

[DMM027680C99] Wu N., Ming X., Xiao J., Wu Z., Chen X., Shinawi M., Shen Y., Yu G., Liu J., Xie H. et al. (2015). TBX6 null variants and a common hypomorphic allele in congenital scoliosis. *N. Engl. J. Med.* 372, 341-350. 10.1056/NEJMoa1406829 25564734 PMC4326244

[DMM027680C100] Xu W., Tan L. and Yu J.-T. (2015). Link between the SNCA gene and parkinsonism. *Neurobiol. Aging* 36, 1505-1518. 10.1016/j.neurobiolaging.2014.10.042 25554495

[DMM027680C101] Yamamoto S., Jaiswal M., Charng W.-L., Gambin T., Karaca E., Mirzaa G., Wiszniewski W., Sandoval H., Haelterman N. A., Xiong B. et al. (2014). A drosophila genetic resource of mutants to study mechanisms underlying human genetic diseases. *Cell* 159, 200-214. 10.1016/j.cell.2014.09.002 25259927 PMC4298142

[DMM027680C102] Zhu H., Lensch M. W., Cahan P. and Daley G. Q. (2011). Investigating monogenic and complex diseases with pluripotent stem cells. *Nat. Rev. Genet.* 12, 266-275. 10.1038/nrg2951 21386866

[DMM027680C103] Zwarts L., Vanden Broeck L., Cappuyns E., Ayroles J. F., Magwire M. M., Vulsteke V., Clements J., Mackay T. F. C. and Callaerts P. (2015). The genetic basis of natural variation in mushroom body size in Drosophila melanogaster. *Nat. Commun.* 6, 10115 10.1038/ncomms10115 26656654 PMC4682101

